# Multidisciplinary marvel: surgical management of aggressive giant cell tumor around the knee in pregnancy: a case report

**DOI:** 10.1093/jscr/rjaf035

**Published:** 2025-02-05

**Authors:** A Ali, B Sahito, S A Irfan, Z Farooq, S Ali, J Iqbal

**Affiliations:** Department of Orthopedics, Dow University of Health Sciences, Karachi, Pakistan; Department of Orthopedics, Dow University of Health Sciences, Karachi, Pakistan; Department of Orthopedics, Dow University of Health Sciences, Karachi, Pakistan; Karachi Medical and Dental College, Karachi, Pakistan; Department of Orthopedics, Chandka Medical College, Larkana, Pakistan; Department of Communicable Disease Center, Hamad Medical Corporation, Doha, Qatar

**Keywords:** giant cell tumor, pregnancy, distal femur, surgical excision, Enneking stage III

## Abstract

Giant cell tumors (GCTs) are rare, representing 4–5% of all bone tumors. Although uncommon in pregnancy, the literature showed the accelerated progression and recurrence of GCT in pregnancy; however, the tumor growth in pregnancy has not been clarified. We report a case of a 28-year-old woman in her first trimester presenting with an aggressive GCT, significantly affecting the patient’s quality of life due to the debilitating nature of her symptoms. Considering the significant functional impairment and aggressive nature of the tumor, a multidisciplinary team opted for surgical intervention, entailing marginal resection and mega-prosthesis implantation. A multidisciplinary approach tailored to the patient’s needs enabled successful surgical intervention and positive maternal and fetal outcomes. This case paves the way for the possibility that surgical management of GCTs can be safely performed during pregnancy, highlighting the challenges and critical importance of multidisciplinary care in rare tumor management during pregnancy through timely intervention.

## Introduction

The giant cell tumor (GCT) was first described by Cooper and Travers in 1818; the GCT is a benign primary tumor of the bone. GCT contributes 15–20% of all the benign tumors of the bone and 4–5% of all bone tumors [1]. The Enneking staging system of tumors categorizes the tumor benign musculoskeletal tumors into latent, active, and aggressive based on the radiographic characteristics of the tumor-host margin. All-demarcated border characterizes the latent lesion, while an active and aggressive tumor has an indistinct border. However, the former is locally or distinctly metastasized [1, 2]. The metastases in locally aggressive benign lesions are rare. However, they are relatively more prevalent in GCT and chondroblastoma.

The literature reported a significant correlation between GCT and pregnancy, suggesting an accelerated tumor progression. The distal femur is the most prevalent site of GCT; despite that, the literature reported GCT as the most frequent tumor in pregnancy in the spine and sacrum [3]. The existing evidence does not have a valid explanation for this unusual correlation; however, the specific oncofetal antigens are held responsible even though the evidence regarding GCT management is scanty in the literature [4]. Formica *et al*. presented detailed literature evidence of 27 cases from 16 studies to address GCT correlation and management in pregnancy. The detailed literature analysis highlights only eight patients with distal femur GCT. However, only four cases were managed surgically during pregnancy [3].

We report a clinically challenging case highlighting a multidisciplinary approach to address the aggressive GCT in the distal femur in a woman in her first trimester planned for wide surgical excision of the tumor.

## Case report

**Figure 1:**
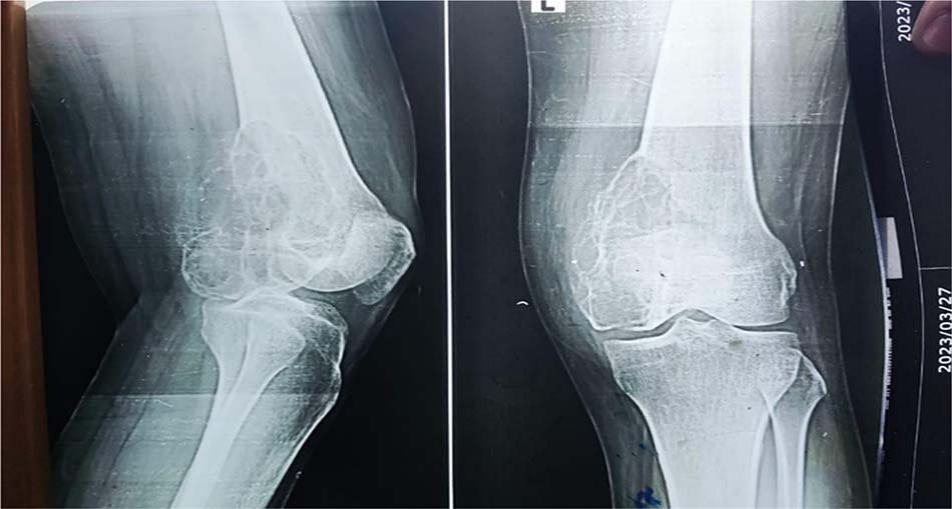
The X-ray shows a distal femur osteolytic lesion with a soap bubble appearance, a narrow zone of transition, a soft tissue component, and a sparing knee joint in the X-ray taken 6 months before pregnancy.

**Figure 2:**
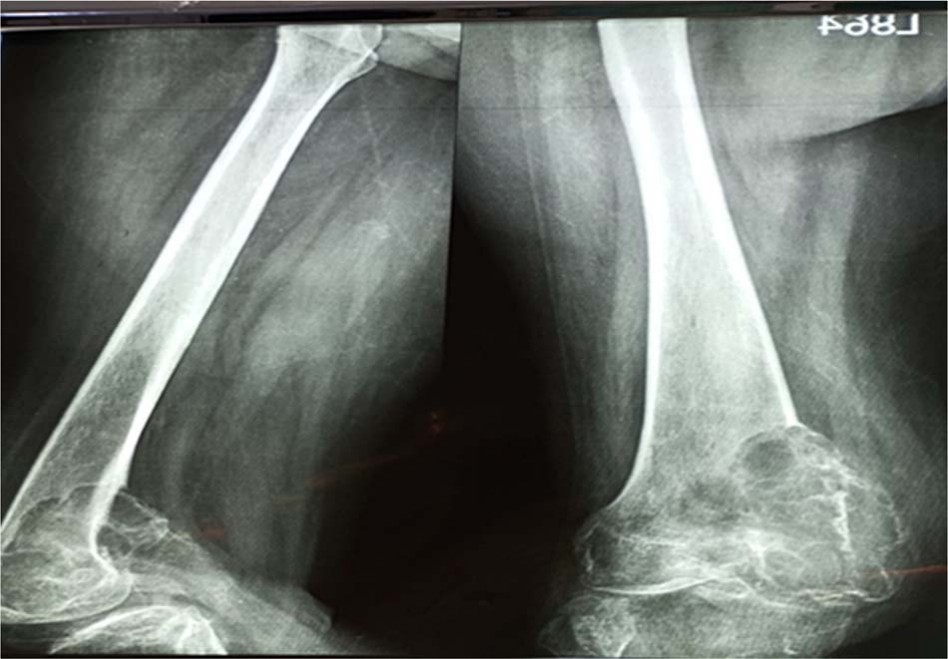
X-ray that was taken 4 months before presentation showing the tumor was very aggressive with a narrow zone of transition, and mass is progressively increased.

A 28-year-old female patient in her first trimester of pregnancy presented in our outpatient department with a complaint of persistent left knee pain for the past two years accompanied by a marked swelling around the left distal thigh region for the past 6 months. She had a previous X-ray that revealed an aggressive, Enneking stage III osteolytic lesion involving the medial femoral condyle, characterized by a shallow bubble appearance and a narrow zone of transition (Figs 1 and 2). A biopsy of the lesion was undertaken 6 months before the initial presentation and showed an aggressive GCT.

**Figure 3:**
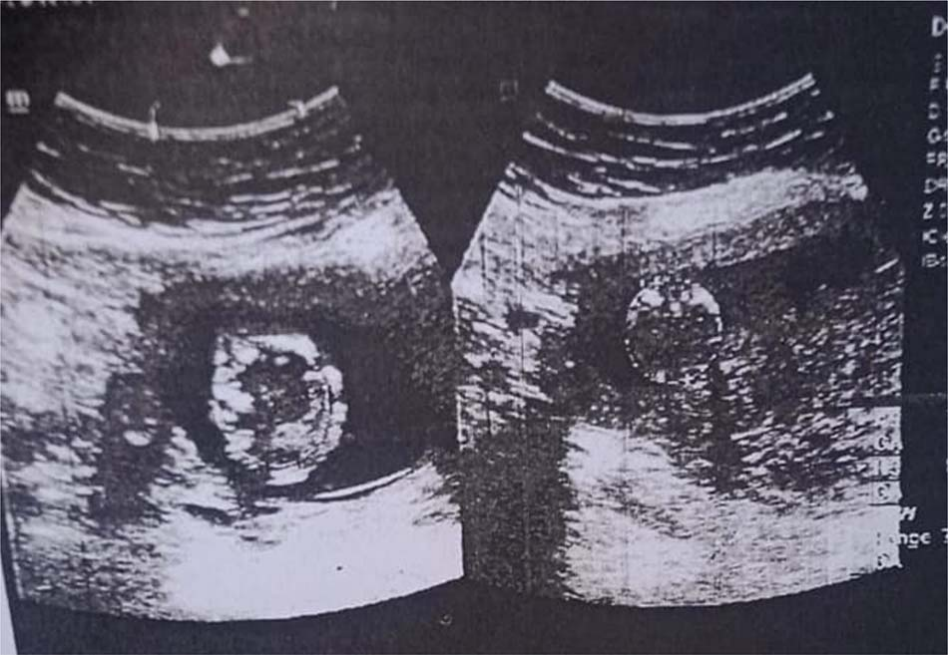
Ultrasound confirmation of pregnancy.

The examination revealed severe difficulty in walking independently. A scar mark measuring 3 cm at the left distal thigh was appreciated, along with a fixed flexion knee deformity of the left knee around 40 degrees. The swelling was firm, non-mobile, and tender on palpation. There was no neurovascular deficit. The obstetric evaluation confirmed the normal fetus status (Fig. 3).

**Figure 4:**
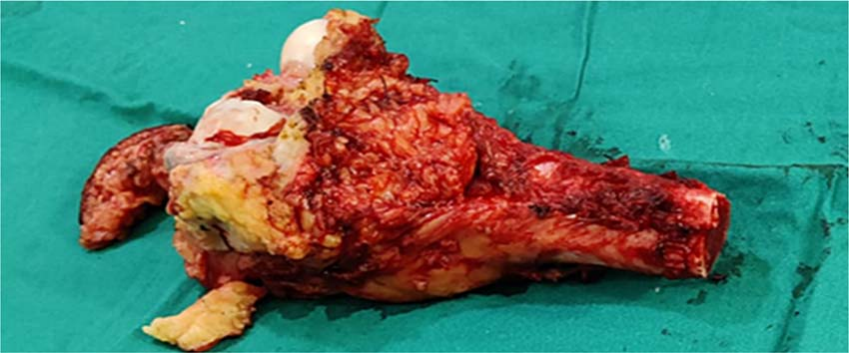
Marginal resected tumor margin and frozen section were taken for confirmation of tumor margin.

**Figure 5:**
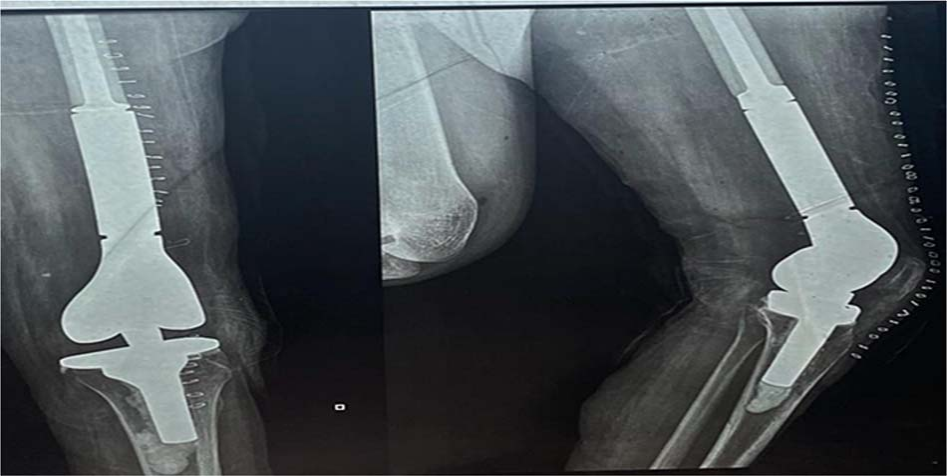
The X-ray after surgery showed a mega prosthesis with good implant alignment.

The functional impairment significantly impacts the quality of life of the patient, and the aggressive nature of the tumor requires surgical intervention. A multidisciplinary team was involved, including orthopeadic surgeons, oncologists, radiologists, and obstetricians. The surgical procedure involved a medial sub vastus approach for marginal resection of the tumor (Fig. 4), followed by a mega-prosthesis knee with 10 mm polyethylene (Fig. 5). Intraoperatively, the tumor was found to be confined to the medial femoral condyle without the involvement of surrounding soft tissues. Alignment was meticulously checked, the wound was closed in layers, a drain was placed, and a knee immobilizer was applied. Denosumab was not used considering the pregnancy.

Postoperative care included continuing antibiotics for two weeks, pain management, and initiating a passive range of motion exercises the next day. The immediate postoperative period was uneventful, and postoperative X-rays confirmed the proper placement and alignment of the prosthesis. The patient was mobilized with the help of physiotherapy, and her mobility gradually improved. She was scheduled for regular follow-up visits to monitor her recovery and the status of her pregnancy.

The long-term prognosis includes regular monitoring for any signs of recurrence or complications related to the prosthesis. The patient was also provided with psychosocial support to help her cope with the stress of her condition and treatment. Following 28 weeks of surgery, the patient delivers a healthy boy via uncomplicated vaginal delivery.

## Discussion

The management of GCTs during pregnancy poses a unique challenge due to the rare occurrence and the complexities involved in balancing maternal health with fetal well-being. The Enneking staging system categorizes musculoskeletal tumors based on radiographic characteristics, helping guide treatment decisions [2]. While GCTs are typically classified as benign, their aggressive behavior and potential for metastasis underscore the importance of early diagnosis and intervention [1, 2]. Vaishya *et al.* and Vanel *et al*. illustrate the need for comprehensive treatment strategies to address both the primary tumor and any associated metastases, emphasizing the importance of a multidisciplinary approach to optimize patient outcomes [5, 6] (Table 1).

**Table 1:** Literature summary of previously published cases reported GCT in the distal femur in pregnancy

**Study**	**Site of GCT**	**Gestational Period at presentation (weeks)**	**Management**	**Time of Surgery (weeks)**	**Outcomes**
Fujibuchi *et al*. 2017	Right distal femur	15	En bloc resection, total knee arthroplasty with endoprosthesis	16th gestational weeks (first surgery), postpartum (second surgery)	Successful management, no complications for mother or fetus; no recurrence after 3 years
Vaishya *et al*. 2006	Right iliac bone, right proximal femur, right distal femur, right patella, right talus	Not specified (initial symptoms during a previous pregnancy, diagnosed postpartum)	Curettage, bone cementing, selective embolization, right patellectomy, curettage and cementing, prophylactic distal femoral locked plate fixation, curettage, and autologous bone grafting	Post-partum	Significant reduction in pain and swelling, pain-free at 1-year follow-up, patient managed daily activities with little assistance
Sharma *et al*. 2006	Left lower femur	18	Curettage, debulking, bone grafting, repeat grafting, therapeutic excision, allografting	29th gestational week	Good recovery, successful delivery of a healthy baby at 39 weeks, uneventful postoperative period for both mother and baby
Vanel *et al*. 1983	Right distal femur	Not specified	Irradiation (40 Gy), biopsy, amputation, resection of pulmonary metastases	Postpartum	Initially, good health postsurgery, pulmonary metastases resected; remains in good health 4 years post initial presentation and 2 years postmetastasis excision

The existing literature suggests that GCTs are less common during pregnancy. However, the aggressive nature of these tumors and the potential for rapid progression necessitate prompt and effective intervention in pregnancy.

The oncofetal antigens are held responsible for the aggressive nature of the tumor during pregnancy [3], and the literature suggests that the expression of estrogen and progesterone receptors contributes to its progression. The immune-histochemical analysis failed to establish the presence of estrogen receptors in GCT cells. However, progesterone receptors were found in 50% of GCT cells [3, 7]. The detailed review of Formica *et al.* failed to establish the cause of the aggressiveness of GCT in pregnancy; however, they recommend establishing a multidisciplinary team for better outcomes [3].

Fujibuchi *et al*. and Sharma *et al*. both emphasize the urgency of surgical management in cases of aggressive GCTs to alleviate symptoms, prevent further deterioration of function, and minimize the risk of complications [8, 9]. Our case aligns with these findings, as the patient presented with significant functional impairment and marked swelling around the left distal thigh, prompting the decision for surgical intervention during the first trimester of pregnancy.

Surgical management remains the cornerstone of treatment for aggressive GCTs, with various approaches described in the literature, including curettage, debulking, bone grafting, and wide excision with prosthetic replacement [10]. Our case utilized a medial sub vastus approach for marginal resection of the tumor, followed by implantation of a mega-prosthesis knee to achieve adequate tumor control while preserving surrounding healthy tissue. This approach aligns with previous studies demonstrating the efficacy of surgical intervention in relieving symptoms and improving functional outcomes.

The decision to proceed with surgery during pregnancy requires careful consideration of both maternal and fetal factors, weighing the risks of surgery and anesthesia against the potential benefits of tumor control. While the literature lacks consensus on the optimal timing of surgery during gestation, our case illustrates the feasibility of performing surgery safely during the first trimester, with the successful delivery of a healthy baby at 39 weeks via normal vaginal delivery.

While non-surgical management options, such as observation or conservative measures, may be considered in select cases, the aggressive nature of the tumor and the extent of symptoms experienced by the patient necessitated surgical intervention [3, 11]. The Enneking staging system categorized the tumor as stage III, indicating an actively growing lesion with a high likelihood of local recurrence and potential for metastasis [2]. Therefore, timely surgical resection was deemed essential to achieve adequate tumor control, alleviate symptoms, and prevent further deterioration of function.

## Conclusion

This case underscores the complexity of managing aggressive giant cell tumors (GCTs) during pregnancy. A multidisciplinary approach tailored to the patient’s needs enabled successful surgical intervention and positive maternal and fetal outcomes. Despite inherent risks, early surgical removal of the tumor was essential due to significant functional impairment and symptom severity. The successful delivery of a healthy baby at 39 weeks and the patient’s favorable recovery highlight the importance of individualized treatment strategies and thorough patient counseling.

## Data Availability

Data is available on request from the authors.

## Guidelines

SCARE guidelines were adopted to prepare the case report [12].
